# Utility of fluorescamine for spectrofluorimetric quantitation of berotralstat hydrochloride in spiked human plasma and content uniformity test and evaluation of method greenness

**DOI:** 10.1038/s41598-025-33306-x

**Published:** 2026-01-12

**Authors:** Hesham Salem, Mohamed Gamal Eldeen, Basmala Sayed, Menna Kamel, Mostafa Embarak, Salma Mokhtar, Hoda Madian, Basmala Bahaa, Sama Alnaser, Mahmoud Abdelgaleel

**Affiliations:** Pharmaceutical chemistry department, faculty of pharmacy, Deraya University, New Minia, Egypt

**Keywords:** Berotralstat hydrochloride, Spectrofluorimetry, Fluorescamine, Content uniformity testing, Greenness, Biological techniques, Chemistry, Drug discovery

## Abstract

A novel, simple and highly sensitive spectrofluorimetric approach has been developed and validated for the first time for determination of berotralstat through derivatization reaction using fluorescamine as a fluorescent probe. The reaction was carried out in buffered medium at pH 7.5, using a borate buffer. After being illuminated at 390 nm, the fluorescence intensity of the resulting product was measured at 480 nm. The proposed approach demonstrated linearity within the concentration range of 100 to 1000 ng mL^− 1^. LOD and LOQ are 6.98 and 26.36 ng mL^− 1^, respectively. Pharmaceutical capsules of berotralstat were successfully evaluated in addition to spiked biological fluid. The statistical data has been validated in accordance with ICH criteria. Additionally, it was applied to examine the content uniformity in accordance with US Pharmacopeia requirements. Furthermore, the results of the ecology scale scores indicated that the analytical process involved was green.

## Introduction

**Fig. 1:**
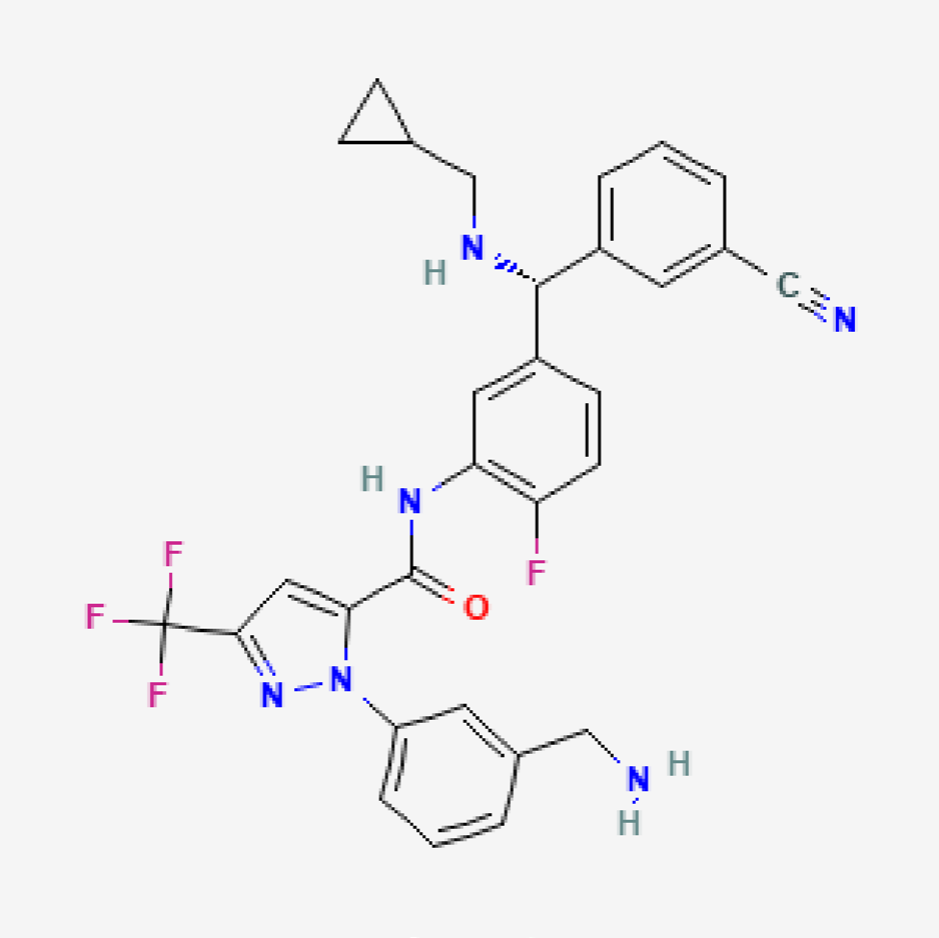
Chemical structure of BER.

Berotralstat (BER) is 2-[3-(aminomethyl)phenyl]-N-[5-[(R)-(3-cyanophenyl)-(cyclopropylmethylamino)methyl]−2-fluorophenyl]−5-(trifluoromethyl)pyrazole-3-carboxamide (Fig. 1). It is a Kallikrein inhibitor approved for hereditary angioedema, which offers suppression of bradykinin-driven glioblastoma progress as it targets localized bradykinin production. It is ideal for repurposing as a treatment adjunct in glioblastoma given its potential to mitigate peritumoral edema, key contributor to morbidity, mediated by kallikrein-induced bradykinin release. Notably, glioblastoma-associated edema and the resultant reliance on corticosteroids are linked to poorer survival outcomes. Berotralstat hydrochloride could address this multifactorial challenge by (i) inhibiting tumour migration, (ii) impeding growth, (iii) reducing edema, and (iv) minimizing corticosteroid dependence, thereby potentially delaying disease progression^1^. Only one LC–MS/MS methods were reported to be used to analyze BER^2^. On the reference compared method, a mixture of acetonitrile, Q water, and formic acid was prepared in a ratio of 70:30:0.1%, respectively. The approach was successfully tested within a linear concentration range spanning from 30 to 1000 ng/mL, The observed percentage recoveries of the active pharmaceutical ingredient from the dosage forms of BER exhibited a range of 97.81% to 103.33% w/w. The limit of detection (LOD) and limit of quantification (LOQ) values for BER were determined to be 0.5 ng/mL and 1 ng/mL, respectively. The technique described is based on liquid chromatography, which necessitates a complex and costly apparatus, uses large amounts of dangerous organic solvents, and involves laborious and time-consuming sample preparation procedures. Furthermore, the techniques were frequently predicated on the use of incredibly costly MS detectors. For the highly accurate and precise quantification of a number of medicinal substances, the spectrofluorometric method offered a straightforward and sensitive approach. It also calls for a basic, manageable instrument. Nevertheless, no spectrofluorometric technique for determining BER hydrochloride was documented. Thus, the current work’s goal is to offer a straightforward and accurate spectrofluorometric technique for determining BER hydrochloride.

The derivative reagent used in the current work is fluorescamine due to its high reactivity toward the main amino group found in BER hydrochloride. A very stable fluorescent derivative was produced when the mentioned medication interacted with fluorescamine. The discovered method offers a number of advantages over the previously described chromatographic method, including being simpler, requiring no complex instrumentation, and not utilizing environmentally harmful organic solvents. The devised approach was employed for the analysis of BER hydrochloride containing pharmaceutical capsules and spiked human plasma after being validated in accordance with ICH requirements^3^. Additionally, the technique was used to verify that these capsules’ contents were consistent with USP criteria.

The reaction between BER and fluorescamine is a condensation reaction where the drug’s primary amine reacts with fluorescamine to form a highly fluorescent product. The proposed analytical protocol involves mixing the drug sample with fluorescamine in an alkaline buffer, such as borate buffer at pH 7.50, and measuring the fluorescence of the resulting product at specific wavelengths (excitation around 390 nm, emission around 480 nm) to quantify the drug.

## Materials and methods

### Instrumentation

A JASCO FP-83 spectrofluorimeter was used to perform the spectrofluorometric measurements. The gadget features a PMT tuned to 400 V and a 150 W Xe-arc light. With a scanning speed of 1000 nm/min, the excitation and emission monochromators had slit widths of 5 nm. Measurements were made using quartz cells with a diameter of one centimeter. Using the Aquatron water still 4000 d (Cole-Parmer, Staffordshire, UK), double-distilled water was created. Also used were the digital balance (Sartorius, Germany), Sonix TV SS-series ultrasonicator (South Carolina, USA), and Jenway 3505 pH meter (Staffordshire, UK).

### Chemicals, reagents and pharmaceutical preparations

All of the solvents and chemicals utilized in this experiment were of analytical grade, with no additional purification was done on them. 20 mg of fluorescamine (Sigma-Aldrich Chemie GmbH, Steinheim, Germany) was blended in 100 mL of acetone to create 0.02% w/v. Acetone, acetonitrile, sodium hydroxide, methanol, ethanol and boric acid were purchased from El-Nasr Pharmaceutical Chemicals Co (Abu-Zabaal, Cairo, Egypt). Utilizing 0.1 M sodium hydroxide (4 g L^− 1^) and 0.1 M boric acid (6.118 g/L) in distilled water, the borate buffer was utilized to span the pH range of 6.5 to 9.0. Berotralstat hydrochloride was obtained from Sigma-Aldrich, a part of Merck KGaA, Darmstadt, Germany and the 150 mg capsules of Orladeyo^®^ were bought from market.

### Standard solutions

An accurate weight of 50 mg BER hydrochloride was transferred into volumetric flask of 50 mL to create the stock standard solution (1 mg mL^− 1^). Next, twenty five mL of methanol was added, mixed well, and the volume was completed with same diluent to the appropriate level. Transferring 0.5 mL of the BER hydrochloride stock solution (1 mg mL^− 1^) into volumetric flask of 50 mL, followed by the addition of same diluent to the mark, produced the working solution (10 µg mL^− 1^).

### Construction of the calibration graph

A series of 10 mL volumetric flasks were filled with 1.2 mL of (0.02% w/v) fluorescamine solution and 1 mL of borate buffer (pH 7.5). Aliquots of the standard BER hydrochloride solutions with concentrations ranging from 100 to 1000 ng mL^− 1^ (produced from their respective working standard solutions, 10 µg mL^− 1^) were used for further translocation. Using ethanol as a diluent, the volume was completed and allowed to sit at room temperature for 10 min in order to acquire the whole reaction. The relative fluorescence intensity (RFI) was measured at 480 nm after excitation at 390 nm. Concurrently, a blank experiment was carried out using the identical protocols, but without the drug.

### Pharmaceutical dosage forms procedures

A total of ten Orladeyo^®^ capsules were evacuated, ground into a fine powder and carefully combined. An accurate weight of the powder equal to 10.0 mg BER hydrochloride was put into 100.0 mL calibrated flasks, and 75.0 mL of methanol was then added. After ten minutes of sonication, the mixture was filled to capacity with methanol and filtered to produce 100 µg mL^− 1^ of BER hydrochloride drug solution after the filtration process. In accordance with the usual analytical process, various sample among the linear range were created and analyzed. The corresponding regression equation was used to calculate the BER hydrochloride concentrations.

### Content uniformity testing

Using a solution taken from a single capsule extraction at a time, the general analytical process was used to analyze BER hydrochloride in commercial capsules. In accordance with the official USP criteria, 10 Orladeyo^®^ capsules were examined separately, and the consistency of their contents was examined^4^.

### Procedure for spiked human plasma samples

Minia University Hospital provided the human plasma samples, which were kept at −20 °C. Before being analyzed, the plasma samples were let to stand at room temperature. A volume of 900 µL of plasma and 100 µL of BER hydrochloride medication were mixed well and then the plasma proteins were precipitated using 2 mL of acetonitrile. The supernatant layer was separated after centrifugation at 5000 rpm for 15 min and then subjected to general analytical procedure^5^. Ethical approval for use of human plasma was obtained from Deraya center of scientific research with an approval number DCSR-09025-80 on 29-9-2025.

### Determination of reaction stoichiometry

Job’s method was used to determine the reaction stoichiometry. Fluorescamine and BER hydrochloride were combined to create two solutions with the same molarity (1.2 × 10^− 4^ M). The spectrofluorometric method’s general protocol was used. The mole fraction of BER hydrochloride was plotted versus the values of ΔRFI.

## Result and discussion

**Fig. 2:**
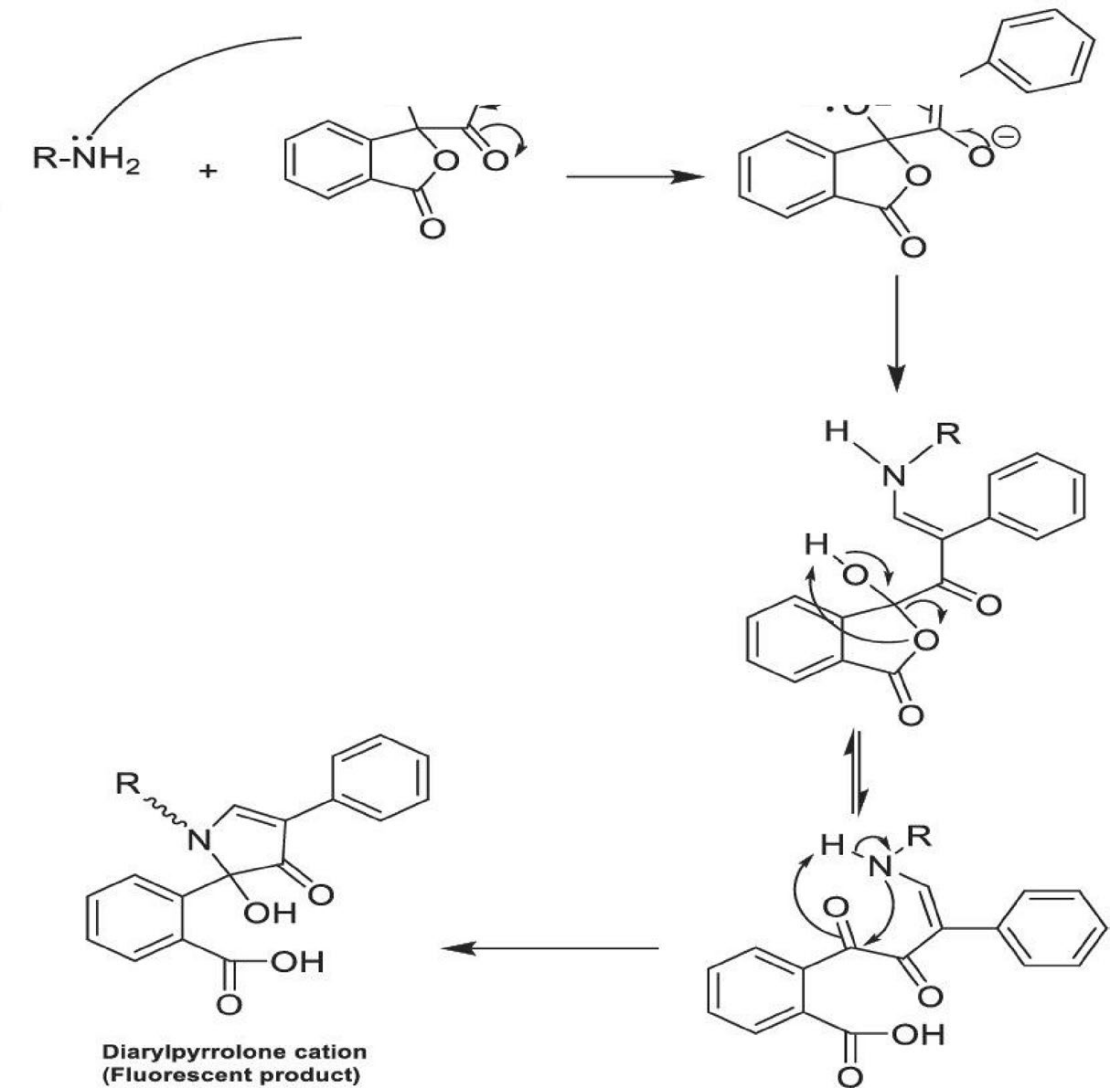
The suggested reaction pathway of fluorescamine and primary amine group in BER.

**Fig. 3:**
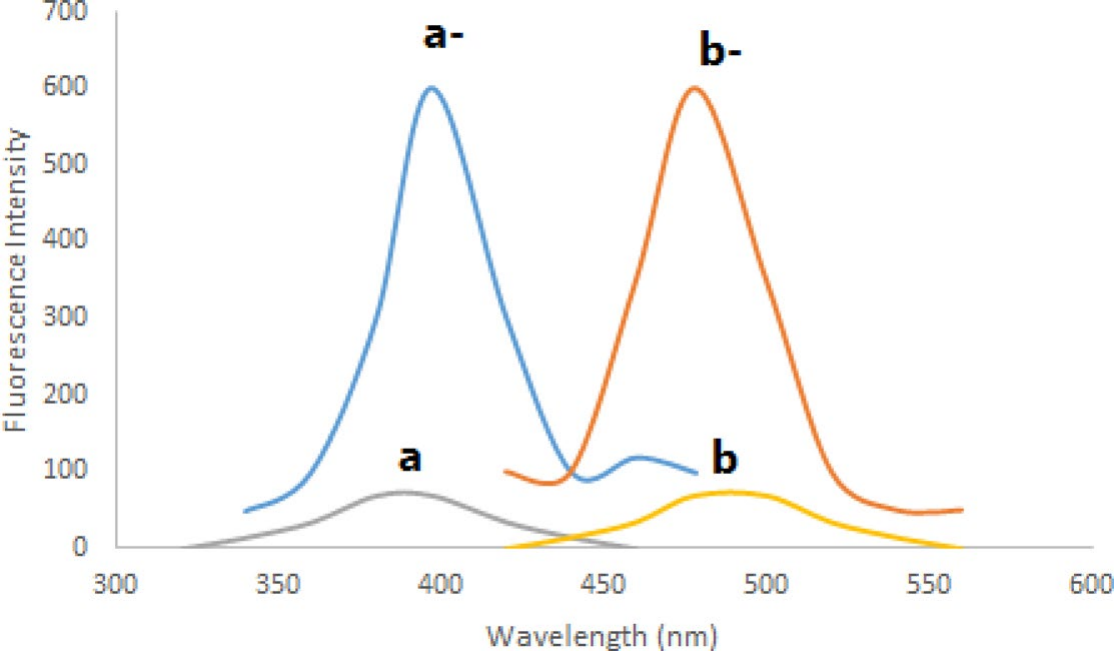
Excitation and emission spectra of 600 ng mL^− 1^ BER-fluorescamine 0.02% reaction product (a^−^ and b^−^) and blank excitation and emission (a and b).

As previously stated, chromatographic techniques were used in every published method for determining BER hydrochloride. However, spectrofluorimetry uses a straightforward tool and process that does not compromise sensitivity or accuracy. Aiding in the development of new eco-friendly techniques is also beneficial in the context of efforts to safeguard natural resources and the environment. Prior to their spectrofluorometric determination, fluorescamine is thought to be a practical and useful reagent for the derivatization of medications with primary amines^6^. BER hydrochloride has a main amino group, which makes it easy to react with fluorescamine reagent in a quick and easy process as illustrated in Fig. 2. This reaction’s highly fluorescent result may be detected spectrofluorimetrically at 480 nm after being excited at 390 nm (Fig. 3). The reaction happens in a buffered medium at room temperature.

### Optimization of reaction conditions

To determine the ideal conditions, the reaction conditions that might have an impact on the fluorophor production or stability were examined. While some of these settings stayed the same, each one was altered separately. pH, concentration of fluorescamine, diluting solvent, volume of buffer, and reaction duration were the factors under investigation (Table 1).

**Table 1:** The selected optimum parameters for the reaction between BER and fluorescamine.

Parameter	Optimum value
pH	7.5
Buffer volume	1.0 mL
Fluorescamine volume	1.2 mL
Diluting solvent	Ethanol
Time	10 min
Molar ratio	1:1

### Influence of pH and buffer’s volume

**Fig. 4:**
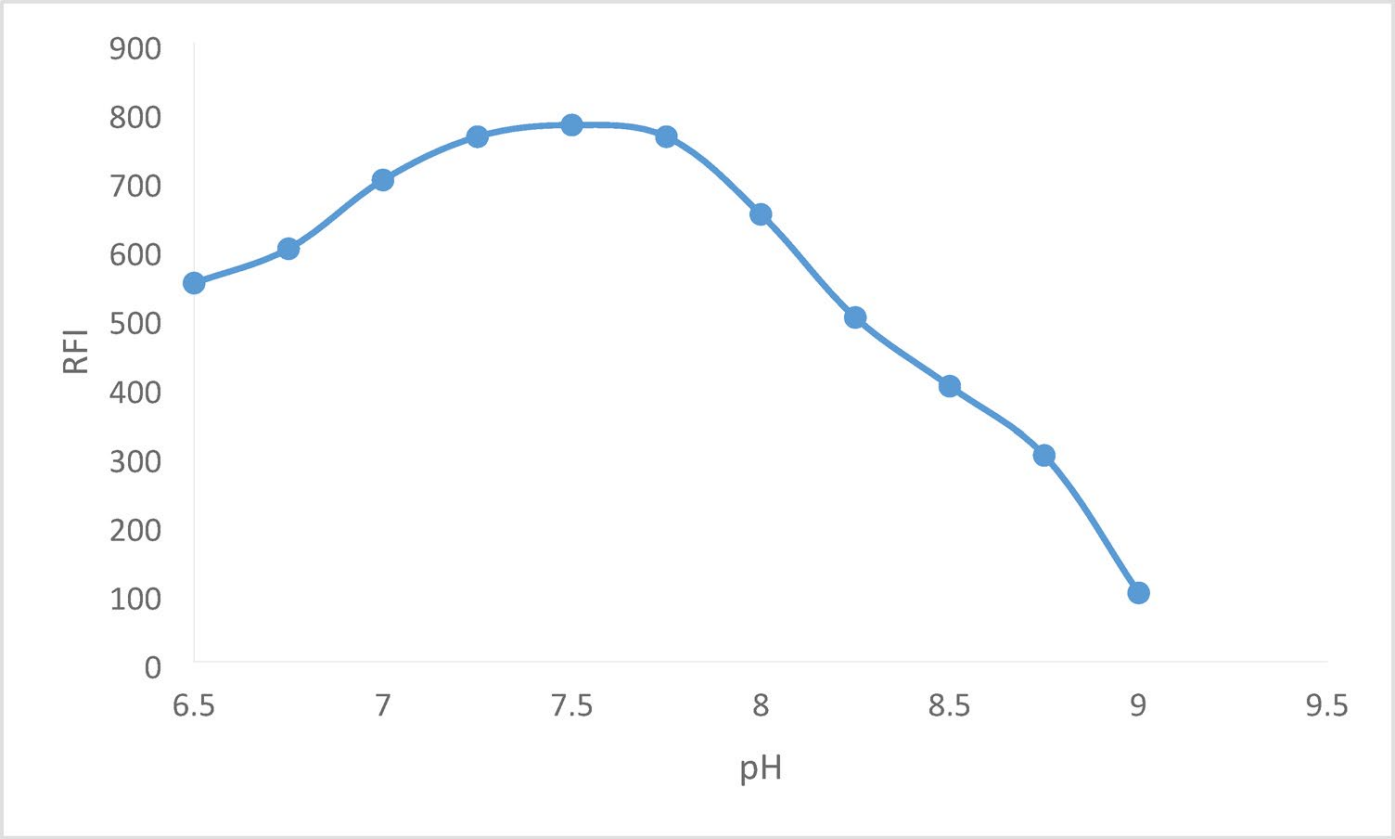
Effect of borate buffer pH on the RFI of the reaction product between 600 ng mL^− 1^ BER and fluorescamine 0.02%.

**Fig. 5:**
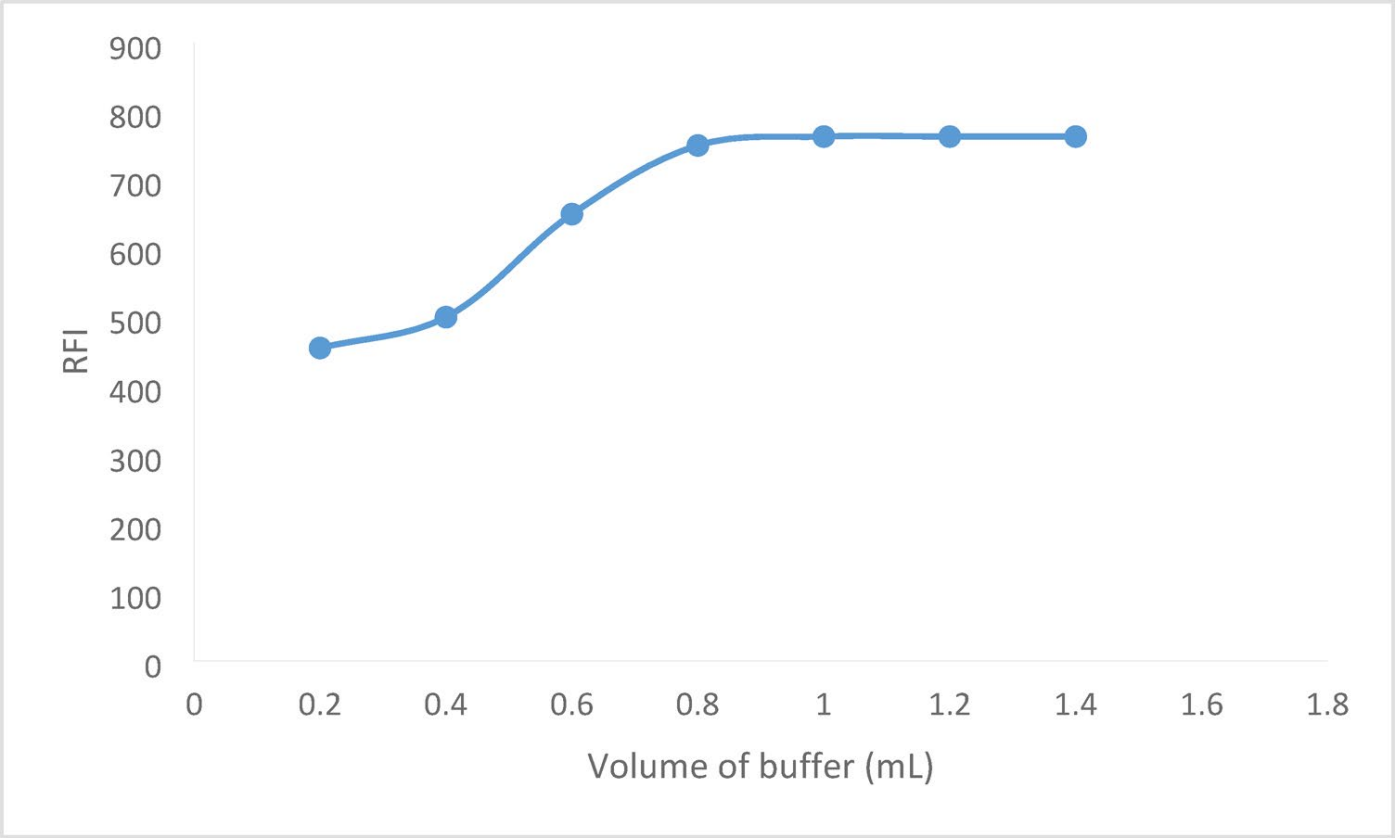
Effect of borate buffer volume on the RFI of the reaction product between BER (600 ng mL^− 1^) and fluorescamine 0.02%.

For the amino group and fluorescamine to react properly, the pH of the solution must be adjusted. Neutral or slightly alkaline media were utilized in the majority of the reported studies in order to accomplish a full reaction and increase the fluorescence of the resultant product. Using borate buffer, the pH range that was investigated in this work was 6.5 to 9.0. The best outcome could be achieved with a pH between 7.25 and 7.75. So, pH 7.5 was chosen as the ideal value (Fig. 4), additionally, various amounts (0.2–1.2 mL) of the borate buffer with a pH of 7.5 were employed to test the impact of a buffer volume. The buffer volume was raised to 1.0 mL in order to boost the obtained fluorescence intensity. The fluorescence intensity plateaued with increasing volume. For the analytical process, 1.0 mL of buffer was selected (Fig. 5).

### Influence of fluorescamine concentration

**Fig. 6:**
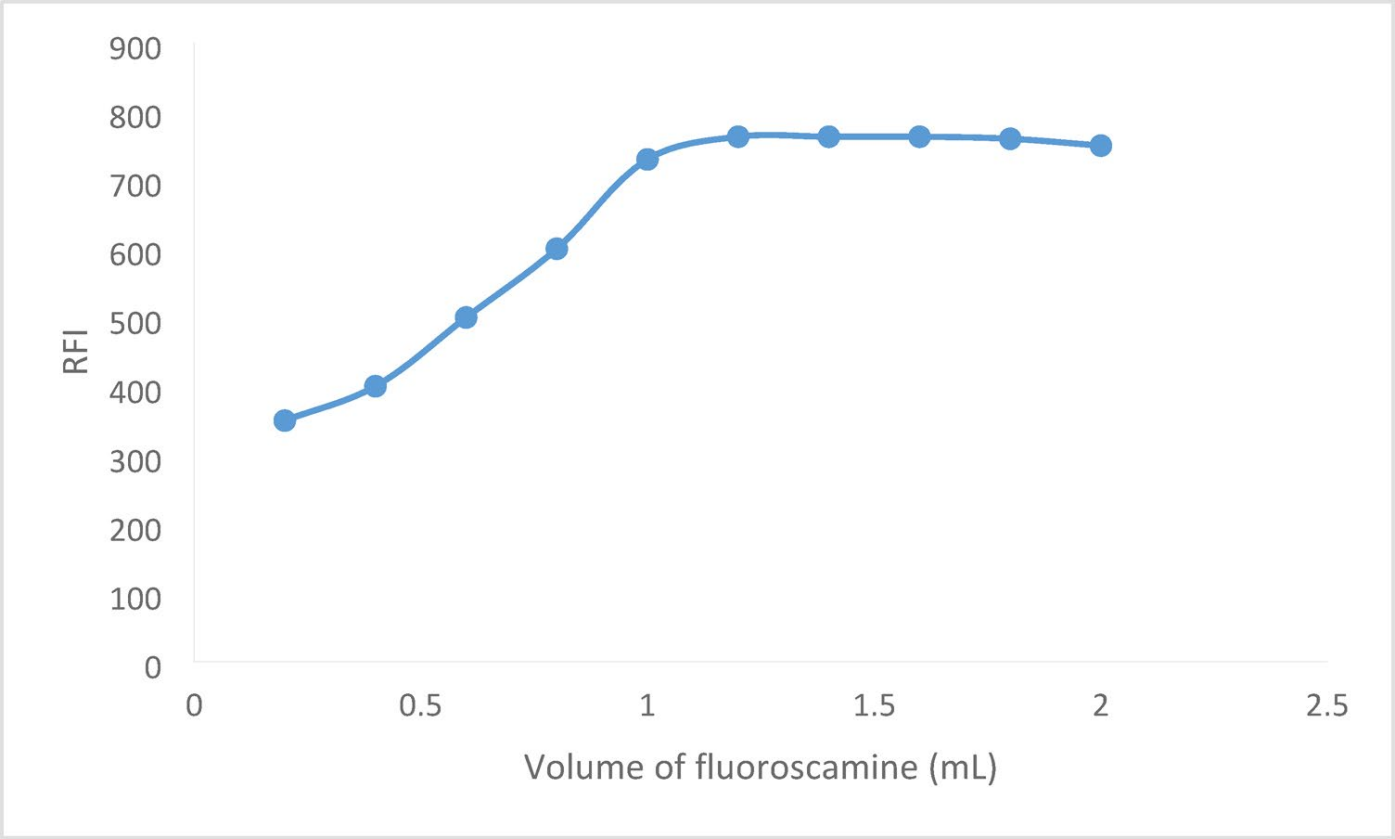
Effect of fluorescamine volume of the RFI reaction product between BER (600 ng mL^−^) and fluorescamine 0.02%.

Using varying quantities (0.2 to 2.0 mL) of fluorescamine solution (0.02% w/v) made in acetone, the impact of fluorescamine concentration was investigated. As the volume of fluorescamine increased, it was observed that the luminous intensity increased as well, peaking at 0.8 mL. The fluorescence values then stabilized(Fig. 6). Therefore, 1.2 mL of fluorescamine was chosen as the ideal volume for more accurate findings.

### Influence of diluting solvent

**Fig. 7:**
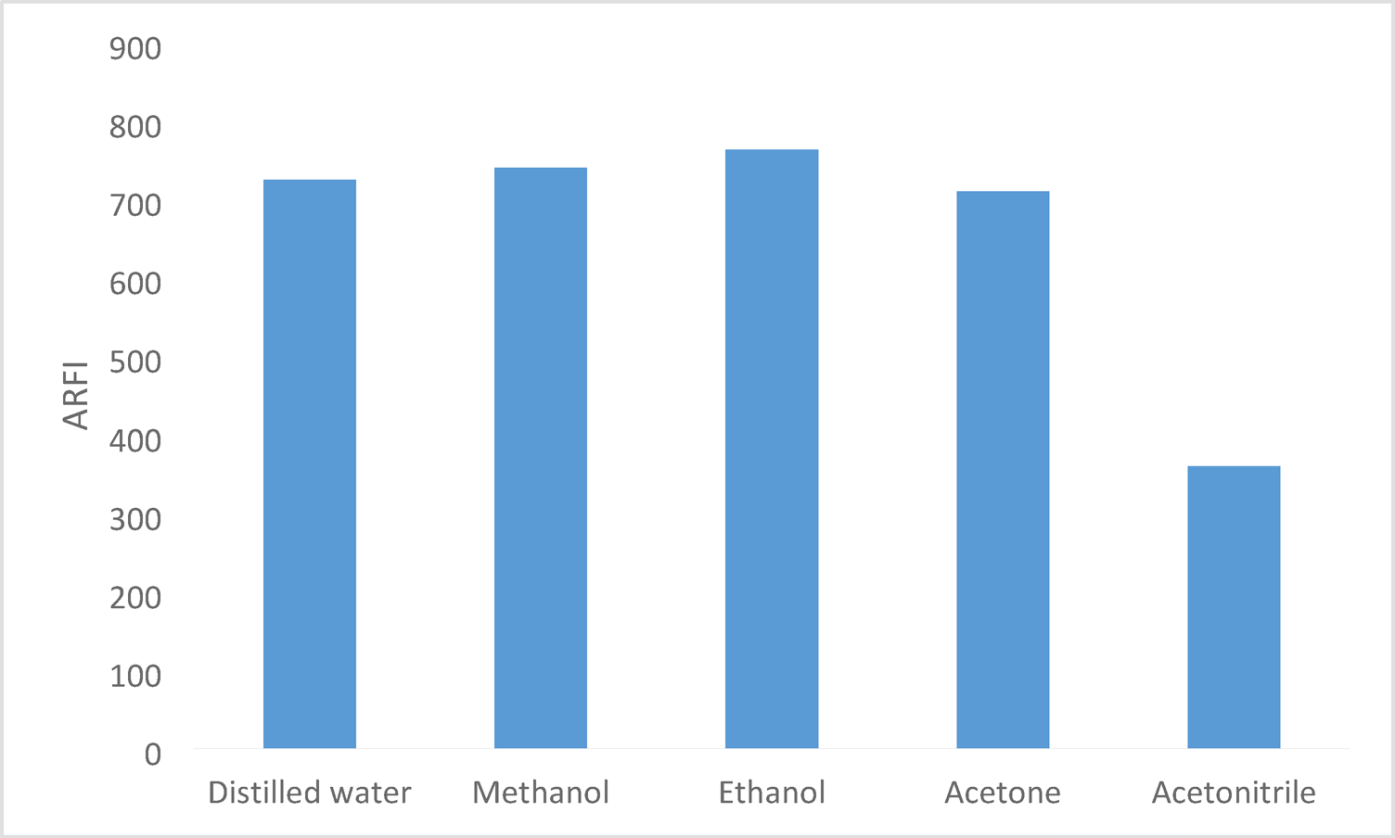
Effect of diluting solvent on the RFI of the product of reaction between BER (600 ng mL^− 1^) and fluorescamine reagent 0.02%.

A variety of solvents, including acetone, acetonitrile, ethanol, methanol, and water were investigated in order to determine which would be the optimal solvent for the reaction. Ethanol was chosen due to its lower toxicity and greater environmental safety than methanol, as acetonitrile produced the lowest fluorescence intensity while ethanol and methanol produced the greatest and most comparable results (Fig. 7).

### Influence of reaction time

**Fig. 8:**
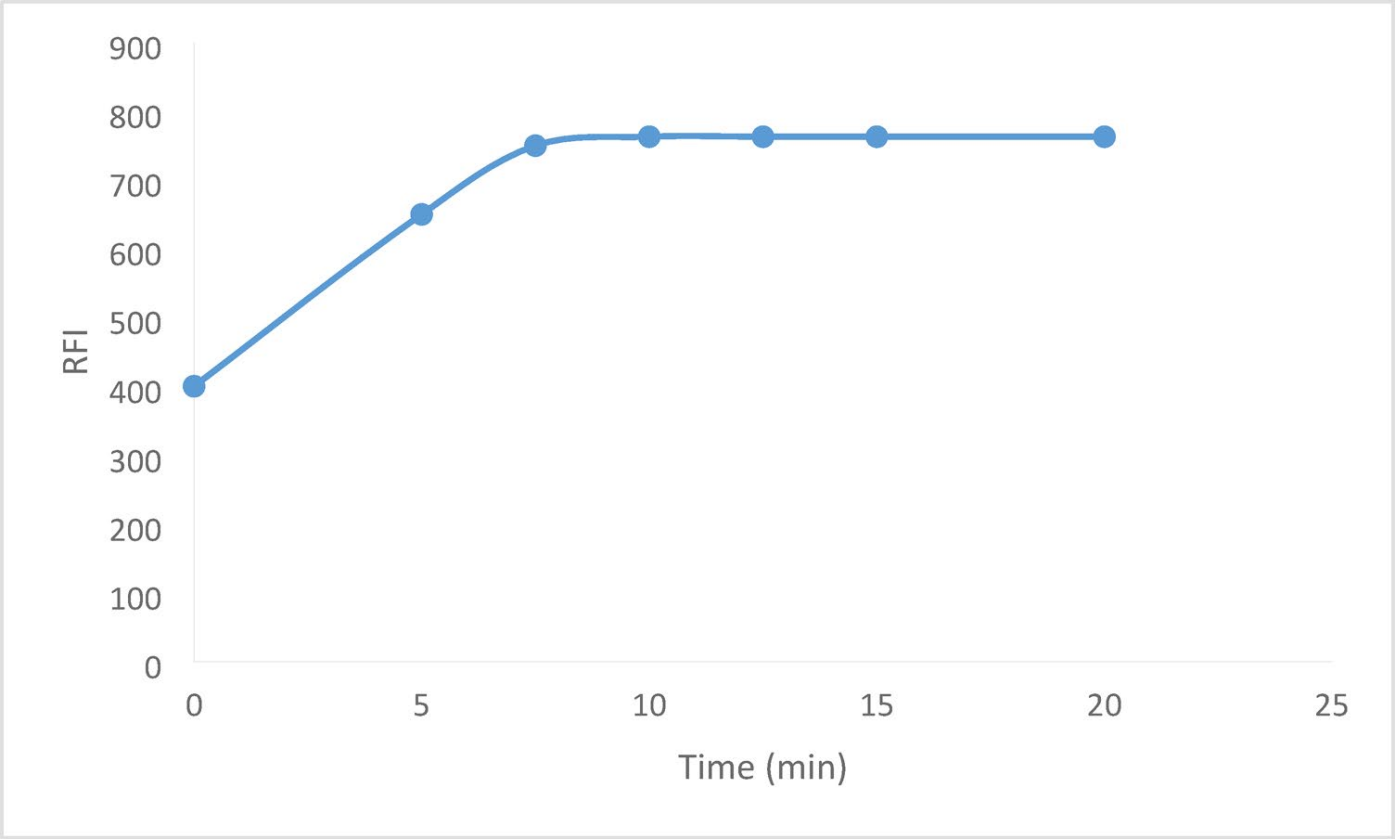
Effect of the reaction time on the RFI of the reaction between BER (600 ng mL^− 1^) and fluorescamine 0.02%.

The medication under study underwent a reaction at room temperature. This gives the suggested procedure an extra benefit. Standing for 10 min produced the highest fluorescence intensity, and standing for up to 20 min had no effect on the reading(Fig. 8). Therefore, the fluorescence intensity was measured in the current work after the solution had stood for 10 min after the reactants had been mixed.

### Determination of the reaction stoichiometry

**Fig. 9:**
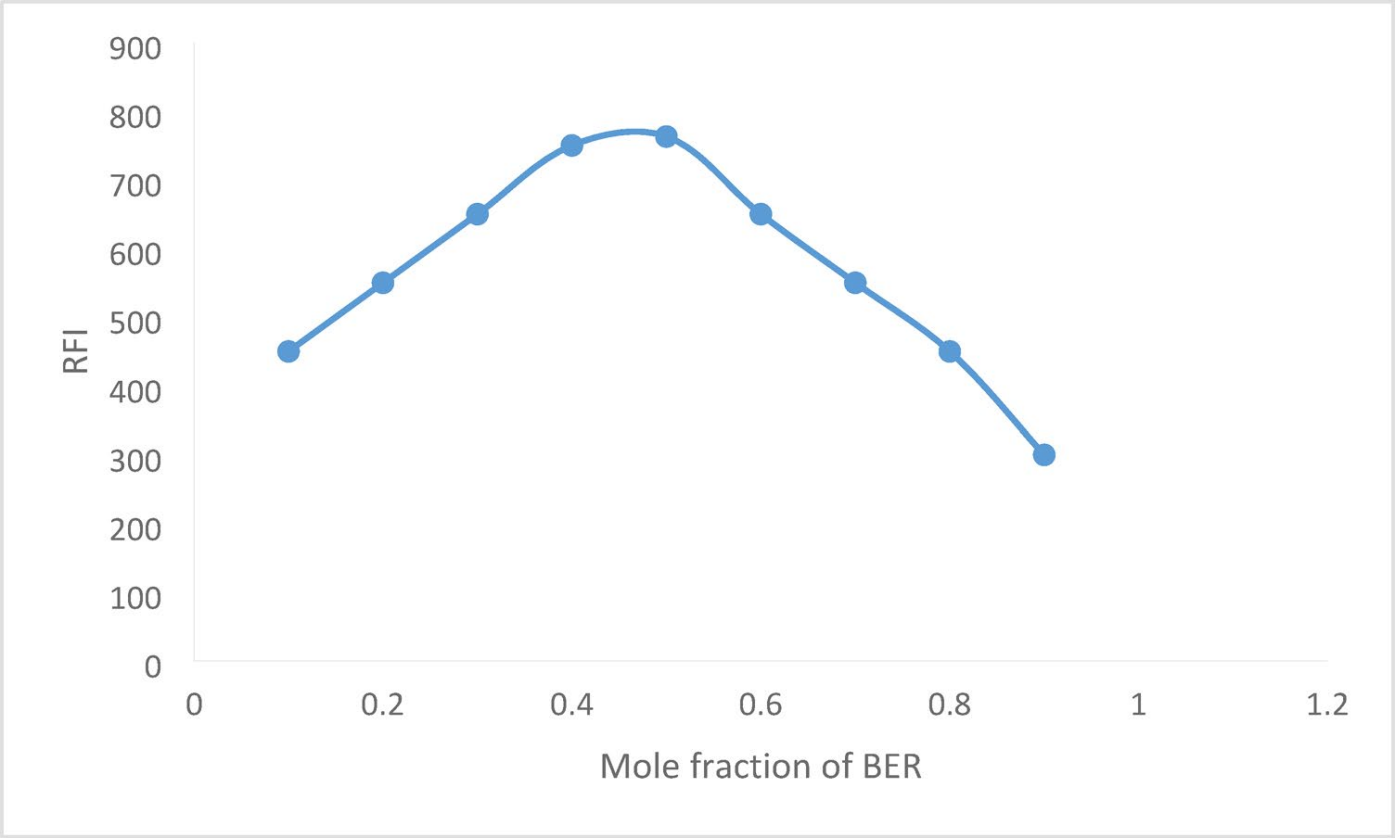
Job`s plot for determination of the stoichiometry of the reaction product using 1.2 × 10^− 4^ M concentration of both BER and fluorescamine.

Job’s approach was used to calculate the stoichiometry of that reaction among BER hydrochloride and fluorescamine. In the usual approach, solutions of both reactants with similar molarities (1.2 × 10^− 4^ M) were created. The fluorescence intensity was correlated with the drug’s mole fraction to create Job’s plot (Fig. 9). The drug’s 0.5 mol fraction exhibited the maximum fluorescence, indicating a 1:1 molar ratio between BER hydrochloride and fluorescamine. The ratio is in line with the specified medication having just one free amino group.

### Method validation

The International Council for Harmonization (ICH) criteria^3^ were followed to validate the proposed technique and evaluate its linearity range, precision, specificity, accuracy, selectivity, LOQ, and LOD.

### Linearity and range

**Fig. 10:**
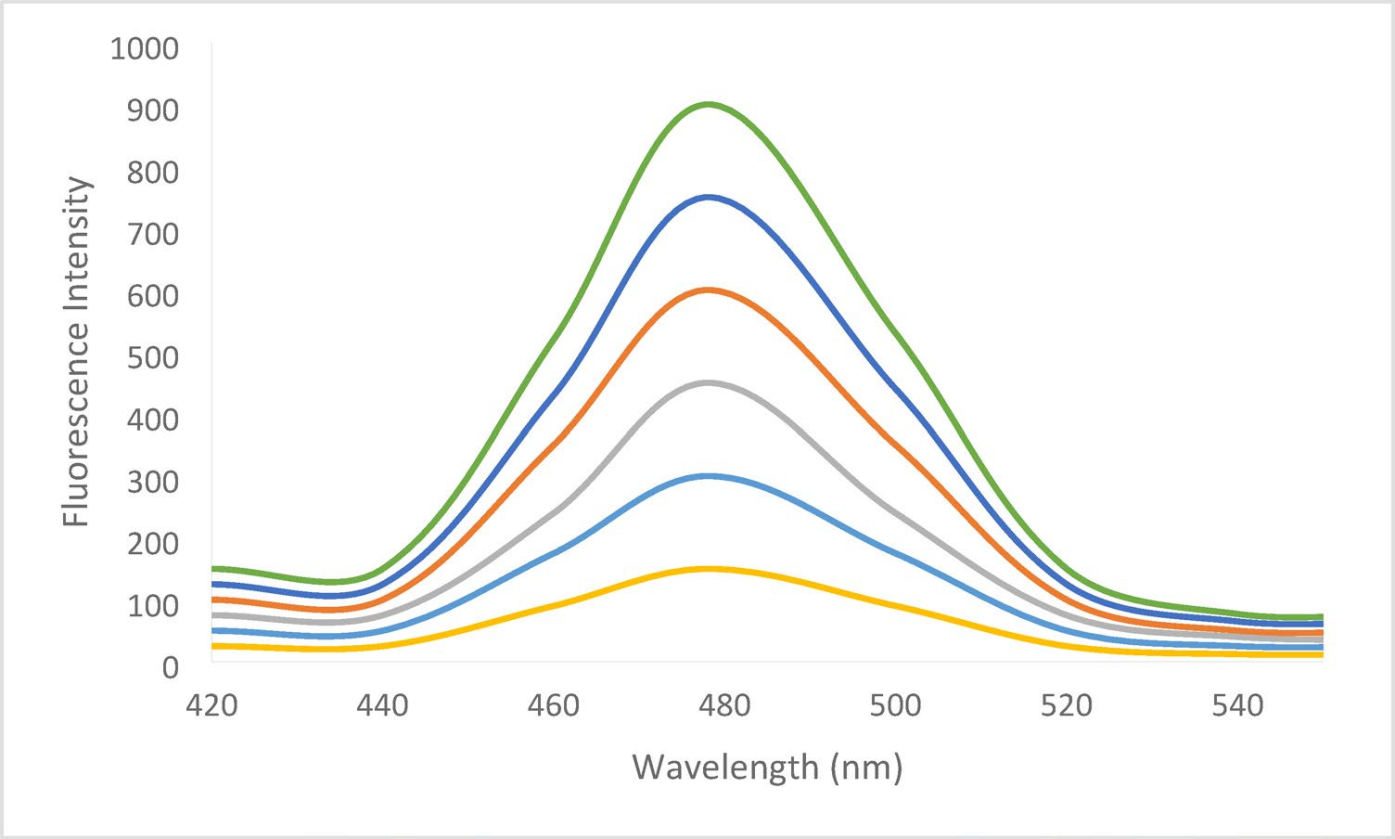
Adding increased concentrations of BER showed a quantitative fluorescence intensity of fluorescamine.

**Fig. 11:**
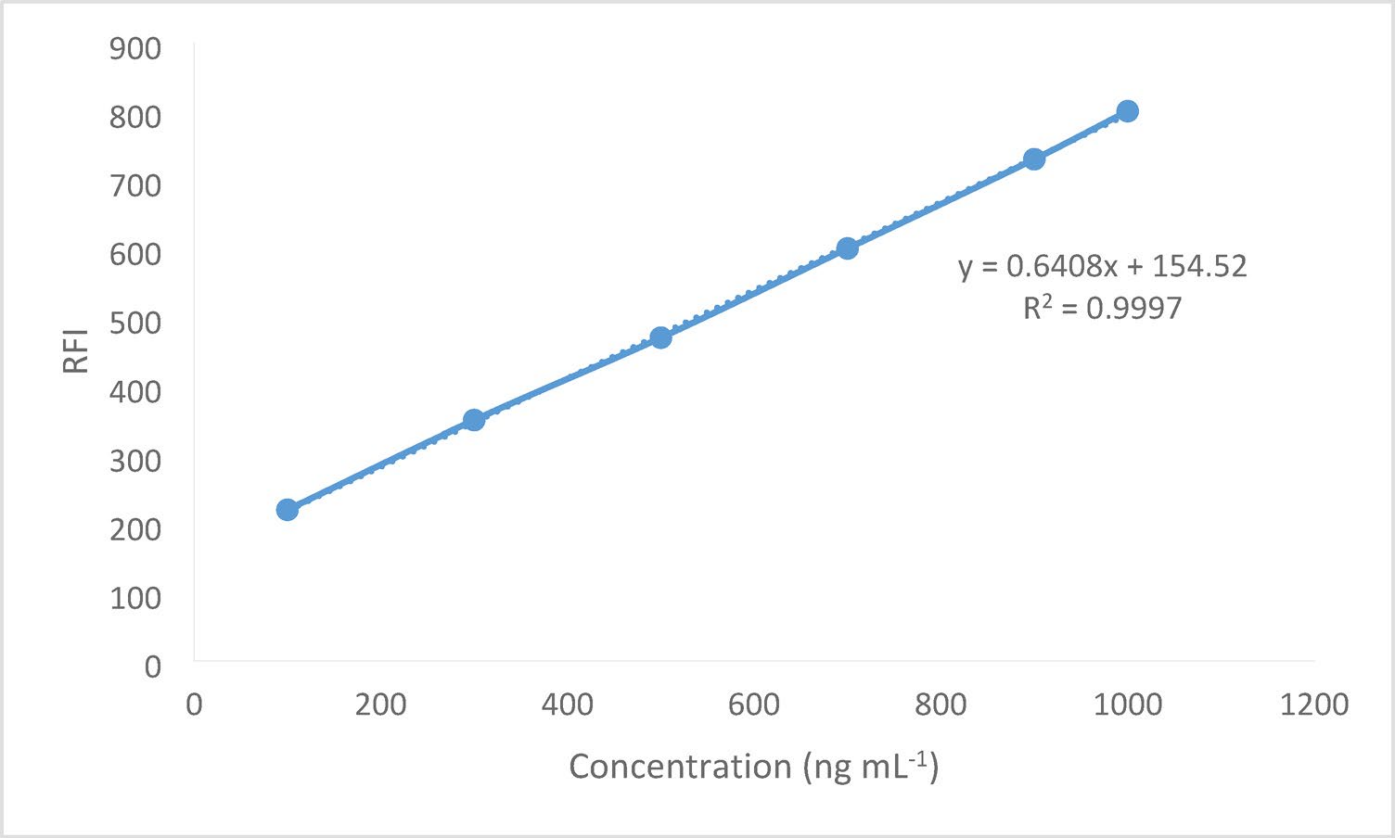
Calibration curve of the suggested spectrofluorometric method for determination of BER via fluorescamine 0.02% as a fluorogenic probe.

Several working medication solutions with different BER hydrochloride concentrations were analyzed using the usual assay protocol after the experimental parameters were optimized. By establishing a correlation between the fluorescence intensity values and the BER hydrochloride concentrations, the calibration plot was constructed. On Fig. 10, adding increased concentrations of BER showed a quantitative fluorescence intensity of fluorescamine. Linear regression equation was applied to statistically process the data and results were listed in Table 2; Fig. 11. The technique demonstrated remarkable linearity with limited scattering of the experimental points which is indicated by the low standard deviation value.

**Table 2:** Analytical parameters for analysis of BER by the proposed spectrofluorimetric method.

Parameter	Proposed method
Excitation wavelength	390 nm
Emission wavelength	480 nm
Linear range (ng mL^− 1^)	100–1000 ng mL^− 1^
Correlation coefficient	0.9997
Slope	154.52
Intercept	0.6410
LOD (ng mL^− 1^)	26.36
LOQ (ng mL^− 1^)	86.98

### Limit of detection (LOD) and limit of quantitation (LOQ)

To assess the fluorescamine method’s sensitivity, LOD and LOQ were computed. The limits were calculated using the following equation, LOD = 3.3 × SD of intercept/slope and LOQ = 10 × SD of intercept/slope. The method showed excellent linearity (100–1000 ng mL^− 1^; *r* = 0.9997) with LOD and LOQ of 26.36 and 86.98 ng mL^− 1^, respectively.

### Accuracy

Six different concentrations lying within linearity range (100, 300, 500, 700, 900, and 1000 ng mL^− 1^) were selected for assessment of the suggested method’s accuracy. For every concentration, three replicate measurements were made. The standard deviation and recovery percentage were computed. Table 3 findings show a close conformity between the computed and real values, indicating the exceptional accuracy of the established approach.

**Table 3:** Accuracy of the proposed method at six concentration levels of BER within the specified range.

Taken (ng mL^− 1^)	% Recovery ± SD
100	100.54 ± 1.25
300	99.38 ± 1.36
500	99.49 ± 0.93
700	100.98 ± 1.39
900	101.53 ± 1.58
1000	99.25 ± 0.99

### Precision

Three different concentrations lying within linearity range (500, 700, and 900 ng mL^− 1^) were selected for assessment of the suggested method’s precision. For every concentration, three replicate measurements were made during the same day and across three days in order to verify the intra-day and inter-day precisions, respectively. The suggested method’s intra-day (repeatability) and inter-day (reproducibility) precision levels were evaluated. Each solution’s RFI was measured, and the regression equation that had been previously computed was used to determine the solution’s concentration. Relative standard deviation and recovery percentages were computed. As shown in Table 4, the acquired percentage RSD values fall within the permitted range.

**Table 4:** The intra-and inter day precision for determination of BER by the proposed spectrofluorometric method.

Concentration (ng mL^− 1^)	% Recovery^a^ ± RSD	
	Intra-day precision	Inter-day precision
500	99.43 ± 1.16	99.27 ± 1.96
700	100.77 ± 0.99	99.29 ± 0.77
900	100.32 ± 0.85	98.99 ± 1.00

^a^ The values are the average of three determinations.

### Robustness

The procedure’s robustness was assessed to determine how well it held up when the experimental variables were slightly changed. Fluorescamine volume, buffer solution volume, and pH were all changed during the trial. Since these little changes in the variables under study had no discernible impact on the established procedure’s outcomes, the data obtained in Table 5 demonstrate the high degree of resilience of the suggested approach.

**Table 5:** Robustness study of the proposed spectrofluorometric method for determination of BER.

Optimization factor	Value	% Recovery^a^ ± RSD
Borate buffer pH	7.4	99.53 ± 1.11
	7.5	100.53 ± 0.99
	7.6	98.49 ± 1.21
Borate buffer volume (mL)	0.9	99.43 ± 1.53
	1.0	99.27 ± 0.67
	1.1	99.30 ± 1.46
Fluorescamine volume (mL)	1.3	100.47 ± 1.85
	1.4	100.90 ± 1.25
	1.5	100.00 ± 1.14

^a^ The values are the average of three determinations.

### Ruggedness

A ruggedness assessment is a method validation process that evaluates how well a test method produces consistent results when subjected to minor variations in conditions, such as different analysts, laboratories, instruments, or days. It ensures the method is robust enough for real-world use and identifies critical factors that can impact results. This is a key requirement for analytical methods, especially in regulated industries like pharmaceuticals. Table 6. Demonstrate the accepted results statistically by three different analysts.

**Table 6:** Ruggedness study of the proposed spectrofluorometric method for determination of BER by three different analysts.

Analyst	% Recovery^a^ ± RSD
Analyst I	100.89 ± 0.98
Analyst II	101.42 ± 1.03
Analyst III	99.99 ± 0.91

## Applications of the suggested strategy

### Application to dosage form

As directed by the general analytical technique, Orladeyo^®^ Capsules were examined. The mean percentage recovery value was 101.68 ± 1.96. In terms of student t-tests and F-tests, the outcomes of the new approach were statistically contrasted with the results using the published chromatographic method^2^. Compared to the tabular data, the computed t- and F-test results were lower. This demonstrated that there was no discernible variation in accuracy and precision among the suggested and published approaches (Table 7).

**Table 7:** Statistical comparison of the results obtained by applying the proposed method and the reported HPLC method for determination of BER in its commercial tablets.

Dosage forms	% Recovery^a^ ± SD		Student’s t-test^b^	F-value^b^
	Proposed method	Reported method		
Orladeyo^®^ Capsules	98.49 ± 1.38	99.30 ± 1.95	0.61	1.05

^a^The values are the mean of three determinations.

^b^The tabulated values of t-test and F test at 0.05% are 2.262 and 5.050, respectively.

### Human plasma application

The standard spectrofluorometric approach was used to test BER hydrochloride in human plasma in order to demonstrate its extraordinary sensitivity. The excellent results for the measurement of BER hydrochloride in spiked human plasma are shown in Table 8. Therefore, without interference from the plasma matrix, the proposed spectrofluorometric approach can be used for the pharmacokinetic analysis of BER hydrochloride.

**Table 8:** Application of the proposed method to spiked human plasma.

Concentration level (ng mL^− 1^)	% Recovery ^a^ ± RSD
500	97.78 ± 1.97
700	98.69 ± 2.85
900	95.09 ± 2.63

^a^ The value is a mean of three determinations.

### Application on content uniformity (CU) testing

When the ratio of active components is less than 25% of total tablet weight or when the drug dose is less than 25 mg, it is advised to verify the consistency of the pharmaceutical dosage units. For the first time, the homogeneity of Orladeyo Capsules units were assessed in accordance with the official USP requirements^4^. Generally, when a complicated analytical process is used, the UC test procedure takes a long time. In contrast, the current method’s process is speedy and straightforward. Consequently, the suggested process is appropriate for this goal. An estimate of the dosage form units’ acceptance value (AV) was made. AV was calculated using the following formula: AV = [M – X] + KS, where S is the standard deviation, K is the acceptability constant, X is the mean of the content for each individual capsule, and M is a reference value. When the AV of 10 pills is less than the maximum permitted acceptance value (L1), the content of the capsule dosage forms is regarded as uniform. The general analytical approach was used to analyze each of the ten Orladeyo Capsules separately.

Each capsule’s content was computed. It was discovered that the acceptance values (AV) for Orladeyo^®^ Capsules were less than the maximum permitted acceptance value (L1), as shown in Table 9.

**Table 9:** Results of content uniformity testing of Orladeyo^®^ capsules using the proposed spectrofluorometric method.

Capsule number	% Recovery of the claimed content of capsules
1	101.79
2	100.99
3	101.54
4	101.38
5	100.63
6	101.96
7	100.85
8	101.00
9	101.64
10	102.64
Mean	101.44
SD	1.59
Acceptance value (AV)	2.75
Maximum allowed AV (L1)	15

### Assessment of the method greenness

Analysts have a huge obligation to protect people and the environment from hazardous chemicals and trash that are produced by their work^7–12^. Green chemistry should be designed and upgraded on a regular basis. The environmental value of the analytical approach was assessed using state-of-the-art considerations, including the Analytical Greenness Calculator (AGREE)^13^ and ecological scale scores^14^. To determine how environmentally friendly the suggested methodology is, eco-scale was used in this study.

The eco-scale evaluation’s findings are shown numerically, with the number of penalty points deducted from 100. The dangers encountered when executing the intended operation are expressed in the points. The greener the process, the higher the score (represented by a high number). There were no extraction or healing procedures in the recommended protocol. Furthermore, for a single sample, the procedure’s energy usage was less than 0.1 k W/h. As a result, the suggested approach has an eco-scale score of 90 (Table 10), which amply illustrates how environmentally sustainable the used strategy is.

**Table 10:** Penalty points calculation for the greenness evaluation of the present method.

Item	Parameter	Word sign	PP score
Technique	Spectrofluorometry	LSH	0
Reagents	Fluorescamine	MSH*	1
Amount of reagent	¬ 10 mL		1
Solvent	Acetone for Flurescamine Ethanol for dilution	MSH LSH	4 1
Heating	No		0
Temperature	Ambient		0
pH	7.6		0
Cooling	No		0
Energy (kWh per sample)	¬ 10		0
Waste	1–10 mL		3
Occupational hazards			0
(TPPs)			10
Eco-scale score	100 - TPP		90

* MSH is an abbreviation for the More hazard, LSH for the less severe hazard and TPPS for the total penalty points.

**Fig. 12:**
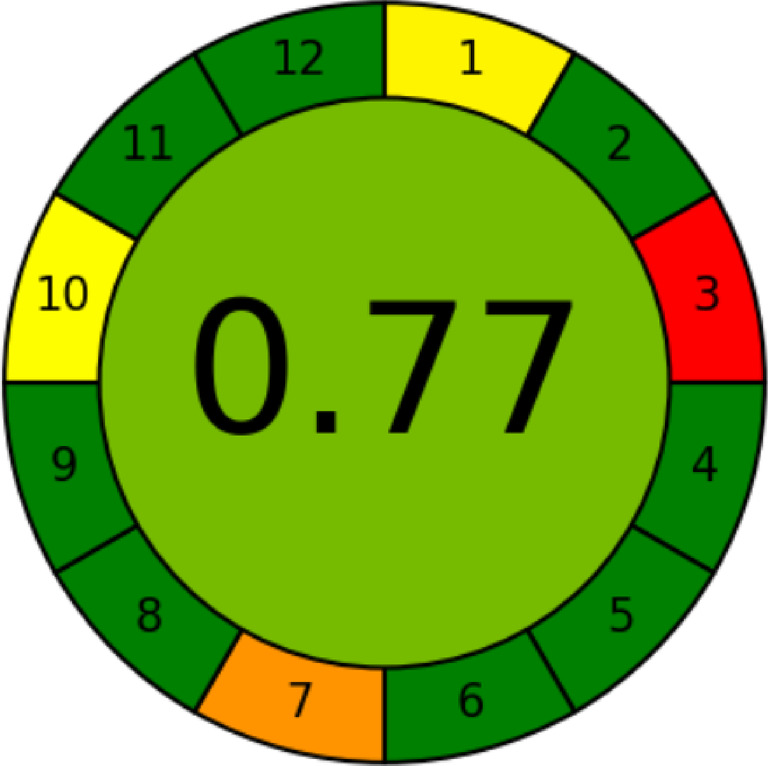
Greenness evaluation of the suggested spectrofluorometric method using AGREE tool.

Green analytical chemistry’s twelve significant principles serve as the input criteria for the user-friendly AGREE program. Each of these twelve inputs is given a score that is shown on an easy-to-understand red-yellow-green color spectrum on a traditional scale ranging from 0 to 1. Additionally, the process considers the importance of each input criterion, which is reflected in the breadth of the corresponding segment. With the overall score and color representation at its center, the output is shown as a clock-like graph. An analysis with a dark green tinge is considered perfect and is given a score of one. The AGREE pictogram (Fig. 12) indicates that the present spectrofluorometric method produced an outstanding green analysis with a score of 0.77.

Penalty points for the greenness evaluation of LC-MS/MS compared method^2^ are typically calculated using a method like the Analytical Eco-Scale, where points are subtracted from a total of 100 based on factors like reagent and solvent amounts, waste generated, energy consumption, and occupational hazards. A higher score (e.g., above 75) indicates a greener analysis. For LC-MS/MS, specific penalties would be assigned for the large amounts of solvents typically used in both the liquid chromatography and the MS parts of the process.

## Conclusion

Based on BER’s interaction with fluorescamine reagent in buffered aqueous solution, this work introduces the first spectrofluorimetric technique for analysis of berotralstat hydrochloride in pure form, pharmaceutical dosage form and spiked plasma. The proposed method is characterized by its simplicity and extraordinary sensitivity. The greenness of the proposed method was assessed and results confirmed the greenness of it. As a result, the developed method is considered a simple, green, and suitable for routine analysis of berotralstat hydrochloride in quality control laboratories.

## Data Availability

All data will be available upon request.
